# Chemotherapy, clocks, and the awareness of death: A quantitative phenomenological study

**DOI:** 10.3389/fpsyg.2023.1097928

**Published:** 2023-03-14

**Authors:** Marcin Moskalewicz, Piotr Kordel, Jadwiga Wiertlewska-Bielarz

**Affiliations:** ^1^Phenomenological Psychopathology and Psychotherapy, Psychiatric Clinic, University of Heidelberg, Heidelberg, Germany; ^2^Philosophy of Mental Health Unit, Department of Social Sciences and the Humanities, Poznan University of Medical Sciences, Poznań, Poland; ^3^Institute of Philosophy, Marie Curie-Sklodowska University, Lublin, Poland

**Keywords:** rhythm, duration of therapy, mental health, mortality, finitude, lived time, cancer, phenomenology

## Abstract

Following a previous phenomenological study of lived time in ovarian cancer, this research aims to find how the frequency of chemotherapy affects orientation in time (the so-called “chemo-clock”) and the awareness of mortality of service users with various cancers. For this purpose, a variation of a front-loaded phenomenological method that combines scientific hypothesis testing with phenomenological insights of both conceptual and qualitative nature was developed. The study is based on a purposive quota sample of 440 participants representative of the Polish cancer population in terms of sex (m:f ratio 1:1) and age (*m* > 65 = 61%; *f* > 65 = 53%) and undergoing chemotherapy for at least a month. The exposure environmental factors of interest are temporal: the frequency of chemotherapy [weekly (*N* = 150), biweekly (*N* = 146), and triweekly (*N* = 144)] and time since the beginning of treatment. The study confirms the relevance of the “chemo-clock”—participants use the pace of hospital appointments for orientation in time, and significantly more often when in triweekly treatments (weekly 38%; biweekly 61%; triweekly 69.4%; V = 0.242, *p* < 0.001, while neither age nor time since the beginning of treatment differentiate the usage of calendar categories and the “chemo-clock”). Simultaneously, chemotherapy increases their awareness of finitude, which again correlates neither with age nor time since the beginning of treatment but is significantly stronger in those with lower chemotherapy frequencies. Lower treatment frequencies are thus associated with its increased significance in terms of its impact on how people with cancer measure time and whether they increasingly consider their mortality.

## 1. Introduction

### 1.1. Three dimensions of time

All life is mortal, but only humans, of all creatures, know that they must die (Jonas, 1996). This knowledge, however, is never permanent. Rather, the awareness of one’s mortality appears fully at rare moments of insight that disclose human finitude (Heidegger, 1962). Such disclosures are unpleasant and inevitably bring about the fundamental existential mood of anxiety.

On the other hand, the cyclic nature of events is the closest substitute for eternity, with which humans can identify. Repetitiveness gives the sense of knowing the past and future; it allows one to feel unlimited and infinite in time. It grounds one’s self in peaceful well-being. A system of internal clocks maintains the recurrent synchrony of organisms with environments (Dawson, 2004). Disturbances of various life cycles and social rhythms studied by chronobiologists, among others, are often associated with health problems (Margraf et al., 2016; Roenneberg and Merrow, 2016). Medicine deals with these disturbances by restoring and maintaining the proper organic rhythms of varying frequencies—cardiac, respiratory, circadian, and menstrual (Haynes et al., 2016; Fuchs, 2018). Social rhythms are also relevant as a measure of cooperation and adherence between clinicians and patients (Thorne et al., 2009).

Time is also an objective, continuous sequence linked to a clock or calendar. It is measured in units such as hours, days, months, and years, which can be used to indicate, e.g., how much of it has elapsed since someone’s birth (age) or the beginning of treatment (duration).

This study situates itself at the intersection of these three temporal dimensions—the awareness of death, the rhythms of medical treatment, and the means of measuring the passing of time. It looks at how cancer treatment cycles affect the patients’ experience of time and their awareness of mortality. More specifically, it aims to asses one of the essential structures of the lived experience of time during chemotherapy in a sequential manner, following the results of a previous qualitative study. The results, as we shall see, are surprising.

### 1.2. Temporal experience in cancer

A diagnosis of cancer alters patients’ experience of time. It presents them with the need to confront death, which affects their lived experience of the future. It also makes their lived time more explicit and focused on the present (Rasmussen and Elverdam, 2007; Khatri et al., 2012; Brown and de Graaf, 2013; Laranjeira et al., 2015). In ovarian cancer, specifically, there is the fear of relapse and the possibility of death (Guenther et al., 2012; Tsai et al., 2020).

Our previous qualitative research concerned a sample of nine women with ovarian cancer undergoing chemotherapy every 3 weeks for at least 6 months (Moskalewicz et al., 2022b). It was a hybrid study that combined a consensual qualitative approach with descriptive phenomenological psychology as developed by Giorgi. The research has uncovered several essential psychological structures underlying how women with ovarian cancer experience time.

One of those structures showed that in a result of their diagnosis and treatment, women with ovarian cancer live time more explicitly. The illness changes how they experience the forward passage of time, which is now burdened with anxieties regarding the possibility of death. It means that not only is the awareness of the passing of time increased, but also it concerns one’s limited lifespan or mortality. The following is an excerpt from a dialogue from the previous study:

Researcher: What has changed the moment you heard your diagnosis?
Participant: A new thinking appeared, how much life do I have left, as far as time is concerned, and existence in general, how much time? It was the key question.

Responding to the same question, another participant answered: And that is it? Is this the end? I used to have the impression that the best is awaiting me, and now it may appear not. Well, it will not most probably appear… Yet another participant simply said: I understood that I am mortal.

None of the interview questions concerned death, but the participants brought this subject up spontaneously. For example: *I thought that you would ask me about my death (…) that you would ask me if I am scared.*

Women with ovarian cancer thus expressed the need to speak about the scarcity of time. This lived temporal structure was named *explicit finitude*. The intense focus of time and increased awareness of finitude and mortality during cancer therapy have also been qualitatively described earlier by others (Velji and Fitch, 2001; Rasmussen and Elverdam, 2007; Khatri et al., 2012; Tindle et al., 2019).

Further on, we have observed that women with ovarian cancer use the triweekly scheme of their chemotherapy sessions as a superior “clock” for orientation in progressing time—a measure that overrides biologically imposed circadian rhythms as well as conventional calendar units, such as weeks (Moskalewicz et al., 2021, 2022b). Participants reported that their life goes in 3-week rounds, which has to do with the subjective significance of their hospital visits. In other words, the treatment rhythm grounds their conceptualization of time, and they live from chemo to chemo. The expectation of the next appointment in 3 weeks was also the guiding event in the participants’ lived temporal horizon.

As one respondent put it: *I live from appointment to appointment. I am not getting excited. I try not to think and to live normally between those hospital visits. When I finished chemo, it was still from appointment to appointment, with no plans*. Another respondent said: *And these trips of mine are kind of, let us say permanently, triweekly, because, God forbid, if I have bad results, everything automatically moves and it is going week by week, and I cannot plan anything important*.

We have termed this phenomenon *chemo-clock*—a symbolic measure of time whose phenomenological value stems from the fact that it was part and parcel of the lived experience of time, even if reflectively objectified (Moskalewicz, 2018). Since the whole sample was subjected to triweekly chemotherapy, we could not have checked whether this phenomenon would also appear in other treatment frequencies. Therefore, we have planned a follow-up quantitative study with participants with various cancers exposed to different chemotherapy frequencies.

### 1.3. Qualitative and quantitative phenomenology

Importantly, our previous qualitative study was phenomenological not only because it utilized Giorgi’s phenomenological psychological method. In addition, the interview questions contained explicitly phenomenological concepts of implicit (pre-reflective) and explicit (reflective) temporality. The subject of temporal experience was thus partly pre-conceived in a phenomenological fashion. At the same time, to minimize our subconscious imputations into the data, we have used the procedure of bracketing our pre-understanding (a version of phenomenological *epoché*). In sum, the previous study aimed to reveal essential structures of the lived experience of time during ovarian cancer chemotherapy. This research presents a quantitative development of the previous findings and its goal is to assess the uncovered temporal phenomena concerning the awareness of death and chemo-clock quantitatively and on a large and more diverse sample of people with cancer. The notion of the *quantitative phenomenological study* indicates that this research directly follows earlier qualitative phenomenological insights, which are now appropriately tested quantitatively.

### 1.4. Concepts of time

This study utilizes three different notions of time:

1. Clock time is a continual objective sequence linked to a clock or calendar. It is measured in units such as hours, days, months, and years. It may indicate how much time has elapsed since the participant’s birth and the start of treatment. We refer to it further as *linear* time.
2. Cyclical time, which is time understood as a rhythm that bases itself off the repetitiveness of various activities in life, such as the frequency of chemotherapy. In the latter context, we refer to it further as *chemo-rhythm*.
3. The awareness of time passing toward death, that is, the explicit consciousness of one’s mortality, which we further refer to as *finitude.*

### 1.5. Hypotheses

We hypothesized that the phenomenon of the chemo-clock stems from the frequency of chemotherapy cycles independently of the duration of treatment and that it would re-appear in the case of weekly or biweekly treatment schemes. In addition, we hypothesized that participants would increasingly consider their mortality during chemotherapy regardless of cancer type and treatment frequency, but these considerations would diminish with treatment duration.

## 2. Materials and methods

### 2.1. Sampling

For sampling, we treated chemo-rhythm as the primary controlled exposure factor of interest and considered chemo-clock and finitude as possible outcomes. Therefore, we targeted three groups equal in size and distinguished them according to three chemo rhythms: weekly, biweekly, and triweekly. For comparison, we were also interested in how linear time affects chemo-clock and finitude. An aspect of linear time (age) was also partly controlled in the sample.

The study used quota sampling based on the most recent (2017) data published by The Polish National Cancer Registry. Each of the three chemo-rhythm groups independently represented the Polish cancer population according to sex (m:f ratio of 1:1) and age (*m* > 65 = 61%, *f* > 65 = 53%). The threshold of 65 years was chosen for two reasons: First, it is the age of retirement in Poland with a likely impact on the lived experience of time. Second, it is the peak of cancer morbidity distribution according to the same data. These quotas correspond to Global Cancer Observatory data (2020) for sex and the estimated age-standardized number of incident cases for females, while for men the sample deviates from the global cancer population data by 4% (*m* > 65 + = 57%, m < 65 = 43%). The further inclusion criterion was undergoing chemotherapy for at least a month (Table 1).

**Table 1:** Sample quotas regarding age and sex in three equal chemo-rhythm groups (total *N* = 440).

			Age < 65		Age > 65		
			Male	Female	Male	Female	Total
Chemo-rhythm	Weekly	*N* (%)	30 (6.8)	34 (7.7)	46 (10.5)	40 (9.1)	150 (34.1)
	Biweekly	*N* (%)	28 (6.4)	34(7.7)	44 (10)	40 (9.1)	146 (33.2)
	Triweekly	*N* (%)	28 (6.4)	34 (7.7)	44 (10)	38 (8.6)	144 (32.7)
	Total	*N* (%)	86 (19.55)	102 (23.18)	134 (30.45)	118 (26.82)	440 (100)
Mean age (years)			52.8	51	70.4	70.1	62.37

### 2.2. Service users validated research survey

We designed a research survey consisting of demographic and background questions (age, time since diagnosis and beginning of treatment, the frequency of chemotherapy, sex, marital and occupational status, place of residence, income, and type of cancer), and statements concerning participants awareness of finitude (formatted in a seven-point Likert scale), questions regarding the measurements of time used during treatment (options: days, weeks, months, chemotherapy, and check-ups sessions), and circumstances in which participants predominantly reflect on the passing of time (options: during or shortly after chemo/shortly before chemo/in other disease-related circumstances/in other non-disease-related conditions/not at all).

To address our potential source of bias regarding the perspective of people with cancer, we invited five patients currently undergoing chemotherapy as experts by experience to edit the survey. To ensure diversity, these were three women and two men, each with different levels of education, including a physician and a psychologist. After including their feedback (mostly having to do with the need to simplify too abstract questions), we conducted a pilot study on a convenience sample of 20 participants with cancer. This survey contained additional control questions assessing the meaning and understandability of the utilized concepts concerning the temporal experience. This was particularly important to retain the meaning of concepts from our previous qualitative study, some of which were phenomenologically burdened. For example, in the final survey, we used the Polish word/concept “przemijanie” (literally: “passing”), which according to the respondents’ feedback, represented the awareness of mortality and the finitude of time. This word/concept is ordinarily used in the Polish language without any cancer experience connotations. However, in the context of the survey, its meaning was understood by the participants as representing what we intended by the concept of finitude. As a result of the feedback received, we developed the final version of the survey. The final question on finitude had the form of a statement: “Since the beginning of treatment, I think about passing more often than before” (measured on a seven-point Likert scale).

### 2.3. Data collection

Data were collected in two main oncological centers in Poznan (where patients from all over Western Poland are treated)—Wielkopolska Oncological Center and the Transfiguration University Clinical Hospital. A paper questionnaire was distributed by nurses working at the chemotherapy wards on behalf of the research team, who were not allowed inside due to the COVID-19 pandemic. The nurses were instructed to approach patients coming to the wards for chemotherapy or check-ups and fitting the inclusion criteria of all the 12 subsamples. The data collection process lasted from October 2020 till May 2021, when the planned total sample size and characteristics was reached. All participants signed a written informed consent form. The project was approved by both institutions’ Independent Review Boards.

### 2.4. Statistical analysis

Data were manually transcribed from paper questionnaires to an SPSS spreadsheet. For statistical analysis of the differences between the groups of participants (demographical variables and three chemotherapy frequency groups), we used *χ*^2^, Kruskal-Wallis H, and Mann-Whitiney U tests. All the tests were run using IBM SPSS Statistics v.22 software.

## 3. Results

### 3.1. Sample characteristic

The total sample characteristic regarding linear time was: mean age 62.37 years (Me 66, IQR 13), mean time since diagnosis 23 months (Me 12, IQR18), mean time in treatment 14.57 months (Me 8, IQR15). The participants had the following cancers: colon 38.4%, lung 7%, breast 29.1%, prostate 1.8%, ovarian 1.4%, pancreatic 5.5%, stomach 5%, kidney 2.3%, liver 1.6%, and other 8%.

### 3.2. Chemo-clock

Regarding the chemo-clock, most participants (55.9%, *N* = 246) measure the passing of time using the pace of hospital appointments (either for chemotherapy or check-ups). The exposure to different chemo-rhythms differentiates the significance of their chemo-clock, with the triweekly scheme being the most frequent (see Table 2). Simultaneously, as far as linear time is concerned, neither age (>65 vs. <65) nor time that has passed since the beginning of treatment (<5, between 5 and 12, and >12 months), differentiates the usage of various calendar temporal categories within the sample. It is the same with all the demographic variables: sex, marital and occupational status, place of residence, income, and most importantly, the type of cancer (*χ*^2^ test, *p* > 0.05). It is solely the frequency of chemotherapy cycles that is statistically significant. Moreover, chemo-clock usage increases with the lower frequency chemo-rhythm, being the highest for the triweekly scheme. As much as 69.4% of participants following chemotherapy triweekly, measure the passing of time with the pace of their treatment and check-ups rather than the calendar units of days, weeks, and months.

**Table 2:** Chemo-rhythm and time measurements.

			Chemo-rhythm			Total
			Weekly	Biweekly	Triweekly	
Time measurements	Months	*N* (%)	6 (4.0)	3 (2.1)	10 (6.9)	19 (4.3)
	Weeks	*N* (%)	63 (42)	25 (17.1)	20 (13.9)	108 (24.5)
	Days	*N* (%)	24 (16)	29 (19.9)	14 (9.7)	67 (15.2)
	Chemotherapy and check-ups	*N* (%)	57 (38)	89 (61)	100 (69.4)	246 (55.9)
Total		*N* (%)	150 (100)	146 (100)	144 (100)	440 (100)

χ^2^ = 51,599, df = 6, Cramer’s V = 0,242, C = 0,324, *p* < 0.001.

### 3.3. Finitude

Regarding finitude, 66.8% of respondents report considering their mortality more often than before treatment (against 14.5% who do not), which confirms our hypothesis from the qualitative study that not only women with ovarian cancer reflect on finitude more often while in chemo.

These thoughts on finitude appear most often during a chemo session and/or shortly after (20.7%), just before the next chemo session (13.2%), and in other circumstances related to the disease (26.4%). Overall, they appear in all those circumstances related to the disease more often (60.3%) than in other situations (10%). However, these considerations of mortality correlate neither with linear time since the beginning of treatment nor with age, which falsifies our hypothesis. They remain regardless of the length of treatment and apparently have to do with entering chemotherapy itself.

Finally, and surprisingly, for it also falsifies our hypothesis, the awareness of finitude (measured on a seven-point Likert scale) increases with the lowering frequency of treatment. Again, the rhythm proves more weighty than the linear time, regardless of its passed “amount” (since birth or since the beginning of treatment). The difference between chemo-rhythms is not huge but statistically significant (see Table 3). The differences between mean values for all three frequencies, weekly (4.853), biweekly (4.986), and triweekly (5.396), represent the difference between “I rather agree” and “I agree.” On the other hand, the median value is “I rather agree” for weekly and biweekly schemes and “I agree” for the triweekly one. Altogether, this data warrant the conclusion that people with cancer extensively reflect on their mortality during chemotherapy.

**Table 3:** Chemo-rhythm and finitude.

	Chemo-rhythm (*N*)	Mean (SD, *M*)
Increased awareness of finitude (measured in seven-point Likert scale)	Weekly (150)	4.853 (1.5604, 5.000)
	Biweekly (146)	4.986 (1.5974, 5.000)
	Triweekly (144)	5.396 (1.6608, 6.000)

Kruskal-Wallis H test χ^2^ = 13,170, df = 2, *p* < 0.05.

## 4. Discussion

### 4.1. The significance of the rhythm

This study is a sequential exploration of previous qualitative phenomenological research results. The previous study established that women with ovarian cancer use the frequency of their chemotherapy as a superior “clock”—they measure the pace of time by their hospital appointments. We have also discovered that they have a heightened awareness of the passing of time and increasingly ponder their mortality. Since time is one of the key organizing factors of life, such temporal alternations seriously impact their mental well-being. These conclusions were limited, however, primarily due to the small and homogenous research sample.

The key finding of the present quantitative exploration of these findings is that the rhythm of chemotherapy is the defining factor of temporal awareness of people with cancer, regardless of their cancer type, demographic data, and linear time that has passed since birth and the beginning of treatment.

First, chemo-clock usage increases with the chemo-rhythm decrease, being the highest for the triweekly treatment plan. This may lead to the desynchronization of people with cancer and their environment. Such desynchronization, like social jet lag or shift work, may be associated with greater health risks and impact their well-being and mental health (Foster et al., 2013; Baron and Reid, 2014).

Second, people with cancer are more likely to ponder their own mortality around treatment sessions, shortly before, during, and after, as well as in other life circumstances related to the disease, which adds further evidence to the fact that they are prone to increased considerations of death due to illness.

However, these considerations of mortality correlate neither with linear time since the beginning of treatment nor with age, which falsifies our hypothesis. They remain regardless of the length of treatment and have to do with entering chemotherapy itself.

Third, the lowering frequency of treatment is associated with increased considerations of finitude. Presumably, users in triweekly chemo-rhythm have more time for recovery after sessions than the other groups. They get to feel better, which on the one hand, increases the felt significance of appointments, but on the other hand, allows them to reflect on their finitude. Clearly, a lower frequency of treatment is associated with its higher personal significance.

This last finding is surprising. It may result from a longer periodicity of treatment, which gives people with cancer more time for thinking and pondering the issues of existence. Hypothetically, those in the more frequent treatment are more focused on its effects, have a more immediate sense of its impact, and are thus more focused on recovery and not on dying. Regardless of the actual effectiveness of chemo, since more frequent treatment is associated with decreased hedonism (Moskalewicz et al., 2022a), those service users could be living in a more intense “survival-mode,” which would also prevent them from pausing and reflecting on their lives.

In addition, if the more frequent chemo worked faster overall, those users would also be getting better and further away from the considerations of mortality. On the other hand, the triweekly users would have much more time awaiting the next session, and thus a much more extended temporal horizon overall, which could foster increased worries of passing away.

As far as psycho-oncology is concerned, the potential clinical relevance of this finding is that service users with cancer in triweekly treatment schemes, regardless of age, cancer type, and treatment duration, should be given additional attention and be intently observed for the signs of decreased mental health resulting from their increased awareness of time and finitude. They could also be prepared to better cope with these thoughts by the medical personnel. However, even if it seems that more frequent cycles are better for the users from the perspective of felt time, it is the clinical picture of cancer and not the awareness of mortality that determines the frequency of chemotherapy.

While awareness of mortality produces anxiety and angst, it also is a source of creativity and, if appropriately managed, can empower one’s outlook on making the most out of life. In this sense, as existential psychotherapists know well, conscious considerations of one’s finitude may be psychotherapeutically explored and bring betterment to mental health (Yalom, 1980)^.^ However, in the context of these considerations being forced by cancer, they are more likely a source of substantial psychological distress. This is why service users with triweekly treatment schemes would most likely require additional psychological support—a hypothesis that further studies should address and verify. It should also be explored if the increased frequency of treatment (regardless of the type of drugs used) translates into service users’ overall well-being in the long run, which is a counter-intuitive hypothesis stemming from the equally counter-intuitive finding of this study.

All in all, this research presents a variation of a front-loaded phenomenological method for studying the lived experience of time. The method combines scientific hypothesis testing with phenomenological insights of both conceptual and qualitative nature and may be applied to validate other dimensions of subjective experience in empirical terms.

### 4.2. Limitations

In terms of quantitative hypothesis testing, this research has several limitations that must be clearly outlined. First, as far as linear time is concerned, it is a cross-sectional study, which means that we approached the participants at a specific point in time since the beginning of their treatment as well as within their current cycle of chemo. The participants thus filled out the survey at different stages of treatment and had only retrospectively assessed several aspects of their temporal experience on a scale. Nevertheless, thanks to the sample characteristics (quotas for sex and age and proportions for the frequency of chemotherapy), the results are partly generalizable, although not as solid as a prospective cohort study. A further limitation is that no biological or pharmacological variables were tested, which would have put the findings in a more clinical context.

### 4.3. Conclusion

The key finding of this research, namely that the lower frequency of chemotherapy is associated with its increased significance, both on how people with cancer measure time and whether they increasingly consider their mortality, should be treated as a point of departure for further studies. It requires exploration and confirmation/falsification in other fields of medicine and possibly beyond the rhythms of cancer treatment.

## Data availability statement

The raw data supporting the conclusions of this article will be made available by the authors, upon reasonable request.

## Ethics statement

The studies involving human participants were reviewed and approved by Poznan University of Medical Sciences IRB. The patients/participants provided their written informed consent to participate in this study.

## Author contributions

MM: conceptualization, methodology, writing original draft, review and editing, funding acquisition, and supervision. PK: conceptualization, methodology, data curation, formal analysis, project administration, and review and editing. JW-B: conceptualization. All authors contributed to the article and approved the submitted version.

## Funding

Data collection funded by The Polish Ligue Against Cancer (Oncogrants III); manuscript completion funded by National Science Center, Poland (No. 2021/42/E/HS1/00106).


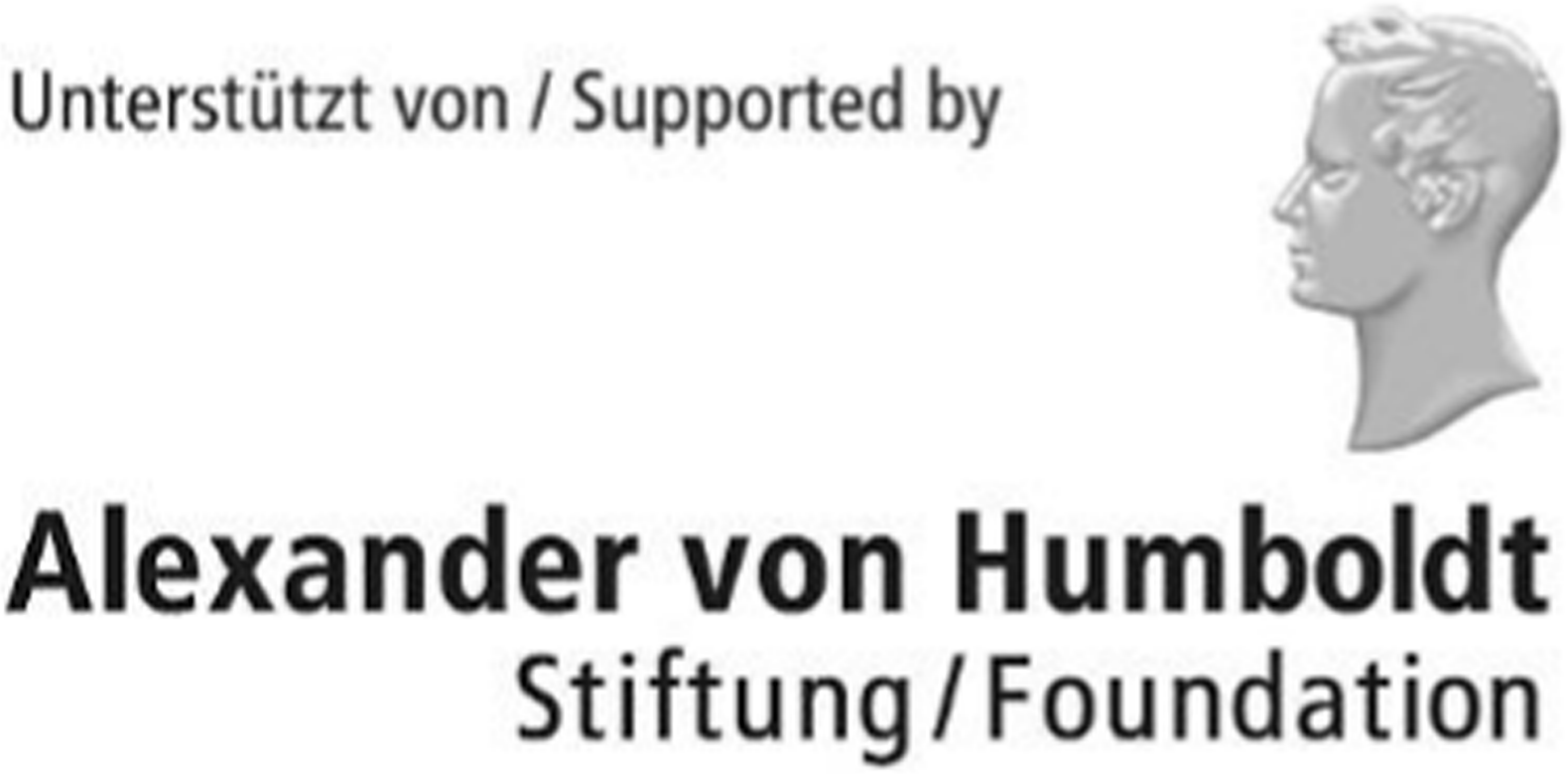


## Conflict of interest

The authors declare that the research was conducted in the absence of any commercial or financial relationships that could be construed as a potential conflict of interest.

## Publisher’s note

All claims expressed in this article are solely those of the authors and do not necessarily represent those of their affiliated organizations, or those of the publisher, the editors and the reviewers. Any product that may be evaluated in this article, or claim that may be made by its manufacturer, is not guaranteed or endorsed by the publisher.
